# Factors associated with postpartum depression in mothers of the kangaroo mother care program in Southern Colombia: a cross-sectional study

**DOI:** 10.1038/s41598-026-45420-5

**Published:** 2026-06-12

**Authors:** Rosa Lisset Salazar Herrán, Gisella Bonilla Santos, Madeleine Melissa Rocha Perdomo, David Steven Sánchez Benítez, Juan David Dussán Chaux

**Affiliations:** 1 Nursing Program, Universidad Surcolombiana, Neiva, Huila Colombia; 2 Psychology Program, Universidad Surcolombiana, Universidad Cooperativa de Colombia, Neiva, Huila Colombia; 3 School of Health Sciences, Universidad Nacional Abierta y a Distancia, Neiva, Huila Colombia

**Keywords:** Postpartum depression, Mental health, Edinburgh’s scale, Kangaroo care, Diseases, Health care, Medical research, Risk factors

## Abstract

Postpartum depression (PPD) is a global public health problem associated with multiple psychosocial and obstetric risk factors. However, evidence remains limited regarding mothers of infants enrolled in kangaroo care programs, a population potentially exposed to increased emotional demands. To establish the frequency and the factors related to postpartum depression in mothers attending kangaroo care in Colombia. An analytical cross-sectional study was conducted using the Edinburgh Postnatal Depression Scale and a structured questionnaire assessing sociodemographic, obstetric-gynecological, and psycho-emotional factors in 140 mothers with infants enrolled in a kangaroo care program. Postpartum depression prevalence was 23.5%. No significant differences were observed across sociodemographic factors. Higher prevalence was associated with vaginal delivery, previous live births, having ≥ 3 children, domestic abuse during pregnancy, and lack of support for infant care. Maternal irritability in response to infant crying was the strongest predictor (OR = 3.64; 95% CI 1.41–9.37), while family support during infant care showed a protective effect (OR = 0.19; 95% CI 0.04–0.88). Identifying the frequency of postpartum depression and its associated factors provides an evidence-based foundation for nursing practice and for healthcare professionals to develop and strengthen postpartum care strategies at the community level. These interventions should integrate maternal mental health and infant care within an interdisciplinary approach.

**Supplementary Information:**

The online version contains supplementary material available at 10.1038/s41598-026-45420-5.

## Introduction

During the perinatal period, women experience profound emotional and psychological changes that increase their vulnerability to mental health disorders, particularly postpartum depression (PPD)^1^.

According to the Diagnostic and Statistical Manual of Mental Disorders, PPD is defined as the occurrence of a major depressive episode within 4 weeks after childbirth, characterized by symptoms such as irritability, excessive crying, and panic^2^. However, accumulating evidence indicates that depressive symptoms may emerge or persist throughout the first year postpartum, extending the period of vulnerability beyond the immediate postnatal phase^3^.

Postpartum depression has significant consequences for both maternal and child health. Affected mothers may experience impaired parenting capacity, diminished enjoyment of the maternal role, and difficulties in establishing healthy mother–infant interactions^4^. These challenges have been associated with early cessation of breastfeeding and reduced caregiving behaviors, which may compromise infant immunity, growth, and development^5^. Moreover, maternal mental health plays a critical role in children’s emotional, cognitive, and psychosocial development, with potential long-term effects across the life course^6^.

Beyond its individual impact, undetected and untreated PPD represents a substantial public health concern. Mothers affected by depressive symptoms may be less able to fulfill caregiving responsibilities, generating broader social and health system consequences^7^. Although postpartum depression occurs worldwide, its frequency and impact vary considerably across social, economic, and cultural contexts^8,9^, reflecting the influence of contextual and structural determinants on maternal mental health.

Previous research has identified multiple factors associated with the onset of PPD, including exposure to stressful life events, a personal history of depression, challenges related to breastfeeding, primiparity, negative body image, and poor partner relationships^10^. Socioeconomic vulnerability has been consistently highlighted as a key determinant, while protective factors such as higher educational attainment, social support, and stable interpersonal relationships have been reported in certain settings^11,12^. These findings underscore the complex and multifactorial nature of postpartum depression.

Importantly, postpartum depression may be triggered by both pre-existing vulnerabilities, such as a prior history of depression, socioeconomic adversity, or exposure to stressful life events, and factors acquired during the postpartum period, including caregiving demands, infant health complications, breastfeeding difficulties, and psychosocial stressors emerging after childbirth^11,12^. This temporal interaction between antecedent and postpartum factors contributes to the heterogeneity of clinical presentations and underscores the complexity of identifying women at higher risk.

Evidence from different world regions suggests that the burden of PPD is unevenly distributed, with higher vulnerability observed among women exposed to social and health-related stressors^13–19^. In Colombia, existing data indicate that postpartum depression remains a relevant maternal health issue, influenced by social conditions and life stressors^20^. However, there is limited evidence focusing on mothers receiving specialized neonatal care, particularly those enrolled in kangaroo care programs, where emotional demands and caregiving challenges may be intensified.

Mothers of preterm or low birth weight infants represent a particularly vulnerable subgroup within the postpartum population. Prematurity and low birth weight are frequently associated with neonatal complications, prolonged hospitalization, and increased caregiving demands, which may intensify maternal emotional distress during the postpartum period. Previous studies have suggested that these conditions are associated with elevated psychological burden, heightened anxiety, and depressive symptoms in mothers, increasing the risk of postpartum depression compared to mothers of full-term infants^26^. Despite this, evidence focusing specifically on maternal mental health outcomes in the context of kangaroo care programs remains limited, particularly in low- and middle-income settings.

Therefore, the present study aimed to identify the frequency and factors related to postpartum depression in mothers attending the kangaroo care program in a high-complexity institution in Colombia.

## Method

### Type of study and participants

Cross-sectional analytical study, conducted in Southern Colombia between August 2021 and May 2022, with 140 women participants who had preterm and/or low birth weight and were in the Kangaroo Care program at the Hospital Universitario Hernando Moncaleano Perdomo. This hospital is the only center that offers the Kangaroo Care program in the city of Neiva, and it is expected that postpartum women will be cared for there with their babies.

Mothers who agreed to participate gave their consent and received a self-administered questionnaire that included the validated Colombian version of the Edinburgh Postnatal Depression Scale- **EPDS.** This instrument is commonly used as a self-administered questionnaire for screening for postpartum depression. In addition, studies have shown comparable efficacy between self-administered questionnaires and face-to-face interviews in diagnosing this condition.

To ensure the data quality, one of the researchers was present at the site of data collection to ensure that the questionnaire was comprehensive.

### Inclusion criteria

Inclusion criteria were NB under 2 months old, registered and active in kangaroo care in a fourth-level health institution and being of legal age.

### Exclusion criteria

Women previously diagnosed with depressive disorder, anxiety disorder, bipolar disorder, addiction to psychoactive substances and/or alcohol, or intellectual disability, were excluded. Besides, it was kept in mind that NBs showed no congenital diseases such as Down’s syndrome, cleft lip, spina bifida, or Edwards’ syndrome.

### Sample size and sampling method

The sample size was calculated using OpenEpi (version 3.0), assuming a finite population of 1000 mothers, a hypothetical prevalence of 12%, a confidence level of 95%, and a margin of error of 5%. The minimum sample size required was 140 participants. Participants were recruited using non-probability convenience sampling based on availability during the study period.

### Instrument

PPD may be associated with some sociodemographic, gynecological-obstetrical, and psycho-emotional factors, which were measured with the following 2-part self-administered questionnaire: (1) Sociodemographic: age, place of residence, socioeconomic status, educational level, marital status, occupation, family and social support. Obstetric and gynecological: number of pregnancies, births, abortions, cesarean sections, live births, stillbirths, smoking, illnesses during pregnancy, difficulty breastfeeding, and perinatal complications (asphyxia, low birth weight). Psychoemotional: planned pregnancy, physical and/or psychological abuse before, during, or after pregnancy, maternal irritability in response to infant crying (MIRIC), adequate fulfillment of the maternal role, and support for infant care (No support, partner support, family support). (2) The Edinburgh postpartum depression scale is a questionnaire consisting of 10 items to explore the mother’s mood during the 7 days before its application, scoring each question from zero to three, according to symptomatic severity and a maximum of 30 points^21^. For Colombia, the EPDS was validated in a study conducted in the city of Cartagena, showing a high internal consistency of 0.78 Cronbach’s Alpha for postpartum depression^22^.

### Statistical analysis

Categorical variables were expressed as absolute frequencies and percentages and compared using Pearson’s chi-squared (*χ²*) test. For ratio-scale variables, the normality assumption was evaluated using the Shapiro-Wilk test alongside visual inspection of histograms and Q–Q plots. As the distribution of these variables deviated from normality, associations between ratio-scale factors (age, gestational age, deliveries, caesarean deliveries, abortions, live births, number of children and number controls prenatal) and ordinal variables (socio-economic status and educational level) with the postpartum depression scale dimensions and total score were assessed using Spearman’s rank correlation coefficient (*ρ*). To identify independent predictors of postpartum depression (PPD), a binary logistic regression model was constructed, with PPD status (Yes/No) as the dependent variable. In accordance with the principles of parsimony and transparency, the initial model included all variables that demonstrated statistical significance (*p* < *0.05*) in the bivariate analysis: vaginal deliveries, live births, number of children, abuse in pregnancy, support for infant care and MIRIC. Model assumptions were rigorously verified: linearity of continuous variables in the logit was assessed via the Box-Tidwell test; the absence of multicollinearity was confirmed using the Variance Inflation Factor (VIF < 5); and independence of errors was validated. Final variable selection was performed through a backward elimination procedure based on statistical significance and model fit optimization according to the Akaike Information Criterion (AIC). All statistical tests were two-tailed, with a significance level set at Alpha (α) < 0.05.

The final model was assessed through internal validation using a non-parametric bootstrapping procedure with 1000 resamples. The 95% confidence intervals (CI) for the adjusted odds ratios (aOR) were estimated using the percentile method. This approach was employed to ensure predictor stability and mitigate potential bias arising from the sample distribution. Data were analysed using JASP statistical software (version 0.19.3).

### Ethical consideration

This study was conducted in accordance with the World Medical Association’s Declaration of Helsinki. Prior to data collection, written informed consent was obtained from participants and the role of the researchers, the objectives of the study, the confidentiality and anonymity of the information, as well as voluntary participation and the possibility of withdrawing from the study at any time were explained to them. Women whose scores indicated a likelihood of postpartum depression, as well as those with a positive response to item 10 of the instrument, would be referred for psychological care at the Hernando Moncaleano Perdomo University Hospital in Neiva, as part of the study’s ethical commitment. Additionally, as a follow-up measure, 1 week after the referral, a psychologist affiliated with the research group would contact the participants by telephone. Approval from the ethics and deontology committee was granted by the Hernando Moncaleano Perdomo University Hospital in Neiva under number 012–005 of 2020.

## Results

Regarding sociodemographic factors, participants reported their age, ranging from 18 to 43 years, with a mean of 27.01 (standard deviation ± 6.44). The Table 1 in the supplementary material shows that most of the participants resided in urban areas, in localities of low socioeconomic stratum, with occupations that do not generate direct income or are unemployed. When comparing the socioeconomic factors, there were no differences between the group of mothers with indicators of depression and the control group. The frequency of PDD was 23.5% in the population studied.

Regarding gynecological and obstetric factors, Table 1 presents the differences identified between the two groups concerning reproductive history. Women with a history of vaginal deliveries exhibited a significantly higher proportion of depressive indicators (30.4%) compared to those without such history (14.8%; *χ²* = 4.67, *p* = 0.031). Similarly, the presence of previous live births was associated with an increase in PPD indicators (37.8% vs. 16.8%; *χ²* = 7.43, *p* = 0.006). Furthermore, the number of children emerged as a relevant factor, as mothers with more than two children presented a higher prevalence of depressive indicators (37.5%) than those with two or fewer children (16.3%; *χ²* = 7.87, *p* = 0.005).

In relation to psycho-emotional and infant-related factors, a history of domestic abuse during pregnancy showed a significant association with the presence of PPD indicators (*χ²* = 6.05, *p* = 0.014); notably, 75.0% of participants who reported maltreatment exhibited depressive symptoms. However, the total number of cases in both groups was low. Infant care arrangements also demonstrated statistical significance (*χ²* = 6.68, *p* = 0.035); women who provided infant care alone reported a substantially higher prevalence of depression (55.6%) compared to those with support from a partner (25.4%) or extended family (17.2%). Finally, MIRIC was revealed as the most robust predictor among the evaluated factors (*χ²* = 11.31, *p* < 0.001). Notably, 46.7% of mothers exhibiting maternal irritability in response to infant crying displayed depressive indicators, compared to 17.3% in the group without this characteristic. The remaining factors exhibited comparable distributions between both groups; detailed data are provided in Supplementary Table 2.

**Table 1:** Gynaeco-obstetric and psycho-emotional factors of mothers attending the kangaroo program.

Factors	Depression indicators				Statistic	*p*-value
	No	%	Si	%		
Gynecological-obstetric						
Number of deliveries						
No	52	85.2	9	14.8	4.67	0.031
Yes	55	69.6	24	30.4		
Live births						
No	79	83.2	16	16.8	7.43	0.006
Yes	28	62.2	17	37.8		
Number of children						
≤ 2	77	83.7	15	16.3	7.87	0.005
≥ 3	30	62.5	18	37.5		
Psycho-emotional						
Abuse in pregnancy						
No	106	77.9	30	22.1	6.05	0.014
Yes	1	25.0	3	75.0		
Support for infant care						
No support	4	44.4	5	55.6	6.68	0.035
Partner support	50	74.6	17	25.4		
Family support	53	82.8	11	17.2		
MIRIC						
No	91	82.7	19	17.3	11.31	< 0.001
Yes	16	53.3	14	46.7		

MIRIC: Maternal Irritability in Response to Infant Crying. The statistic for comparing the factors was Pearson’s chi-squared (*χ²*) test.

### Spearman rank correlation analysis between socio-demographic, obstetric variables, and depression dimensions

Table 2 presents the Spearman’s rank correlation coefficients between the participants’ socio-demographic and gynaecological-obstetric characteristics and the dimensions of the Edinburgh Postnatal Depression Scale. The analysis revealed significant associations, predominantly of weak-to-moderate magnitude, between several risk factors and depressive symptomatology.

Regarding socio-demographic factors, educational attainment emerged as the factor with the most consistent and robust negative association, showing significant correlations with depressed mood (*ρ* = − 0.27, *p* = 0.001), feelings of hopelessness and guilt (*ρ* = − 0.23, *p* = 0.006), depression level (*ρ* = − 0.20, *p* = 0.020), and the overall depression diagnosis (*ρ* = − 0.21, *p* = 0.013). Socio-economic status (SES) exhibited a negative correlation and approached statistical significance only within the depressed mood dimension (*ρ* = − 0.17, *p* = 0.047), suggesting that lower educational and socio-economic levels are linked to a higher symptomatic burden. Maternal age showed a weak but significant positive association only with the depression level (*ρ* = 0.17, *p* = 0.044).

In terms of gynaecological-obstetric factors, a history of deliveries and previous live births were positively correlated with depressed mood (*ρ* = 0.19, *p* = 0.027 in both cases) and with the depression level (*ρ* = 0.21, *p* = 0.013 and *p* = 0.19, *p* = 0.024) respectively). Similarly, the total number of children showed a significant relationship with the depression level (*ρ* = 0.19, *p* = 0.027). Factors such as the number of pregnancies, caesarean sections, abortions, antenatal check-ups, and gestational weeks did not reach statistical significance in relation to the evaluated dimensions of depression (*p* > 0.05).

**Table 2:** Correlations among factors with the dimensions of the post-partum scale (Edinburgh) of mothers attending the kangaroo program.

Factor	Depressed mood	Anhedonia	Hopelessness and guilt	Depression level	Depression
Age	0.12	0.15	0.03	0.17*	0.08
SES	− 0.17*	0.06	− 0.04	− 0.03	− 0.03
Schooling	− 0.27**	− 0.01	− 0.23*	− 0.20*	− 0.21*
Pregnancies	0.08	0.11	− 0.03	0.11	0.02
Deliveries	0.19*	0.02	0.02	0.21*	0.07
Caesarean deliveries	− 0.01	0.10	− 0.00	− 0.03	0.01
Abortions	0.08	0.11	0.12	0.10	0.12
Live births	0.19*	0.13	0.03	0.19*	0.10
Number of children	0.17	0.10	0.02	0.19*	0.07
Number controls prenatal	− 0.08	− 0.13	− 0.08	− 0.06	− 0.10
Gestational age (weeks)	− 0.08	0.09	0.01	0.06	− 0.03

SES: Socio-economic status. Coefficients shown are Spearman’s rho ().

Significant values are in bold.

*p < 0.05. **p < 0.01. ***p < 0.001.

### Logistic regression for the prediction of depression

A multivariate analysis was conducted using binary logistic regression. The dependent variable was postpartum depression, as measured by the Edinburgh Postnatal Depression Scale (EPDS). Independent variables included factors that demonstrated statistical significance in initial bivariate analyses using Pearson’s chi-square (*X*^*2*^) and Spearman’s correlation coefficient. Parity, live births, abuse during pregnancy, age, socioeconomic status, and educational level were excluded during the backward elimination process, as they did not improve model fit based on the AIC; model specifications are detailed in Table 3 of the supplementary material.

The overall model evaluation indicated a significant improvement in fit compared to the null model (likelihood ratio test: Δ*x*^*2*^^4^= 20.51, *p* < 0 0.001). Goodness-of-fit indices corroborated the appropriateness of the proposed model, with the AIC decreasing from 154.91 to 142.39. Regarding explained variance, the model achieved a Nagelkerke *R*^*2*^ of 0.205 and a Tjur *R*^*2*^ of 0.163, suggesting a suitable effect size for the clinical context under study.

Prior to coefficient interpretation, the assumption of non-multicollinearity was verified. Collinearity diagnostics showed tolerance values exceeding 0.88 and Variance Inflation Factors (VIF) below 1.13 for all predictors (see Table 4 of the supplementary material), thereby ruling out multicollinearity issues among the independent variables.

Table 3 presents the estimated coefficients, standard errors, and odds ratios (OR) for the model. Two significant risk factors and one conditional protective factor were identified. Firstly, MIRIC emerged as a significant positive predictor; the presence of this trait was significantly associated with an increased likelihood of depression (Wald = 7.17, *p* = 0.007). Mothers of infants with this condition were 3.64 times more likely to experience depression compared to the reference group (*OR* = 3.64; 95% CI [1.41, 9.37]).

The second variable was the number of children, where the category of having ≥ 3 children acted as a significant risk predictor (*p* = 0.022), increasing the probability of depression by a factor of 2.77 (*OR* = 2.77; 95% CI [1.16, 6.65]). Lastly, infant care showed a significant protective effect only for the category where participants reported receiving help from other family members; this was associated with an 81.3% reduction in the probability of postpartum depression (*OR* = 0.19; 95% CI [0.04, 0.88]). The category indicating exclusive help from the partner did not reach statistical significance (*p* = 0.096).

To validate the stability of the estimates, a bootstrapping procedure with 1000 resamples was employed. The bias-corrected and accelerated (BCa) 95% confidence intervals confirmed the direction and significance of MIRIC (95% CI [1.32, 9.40]) and having ≥ 3 children (95% CI [1.15, 6.29]), as the intervals did not cross the unit. However, it is noteworthy that under bootstrap analysis, the protective effect of receiving help from both partner and family yielded a confidence interval that approached unity (95% CI [0.042, 1.003]). This suggests that the significance of this specific predictor may be sensitive to sample characteristics; thus, caution is advised regarding its generalisability, despite its significance in the Wald test.

**Table 3:** Multivariate analysis between factors with post-partum depression in mothers attending the kangaroo program.

Predictor	Estimate	Standard error	Wald	*p*	OR	CI 95% Wald	CI 95% Bootstrap
*(Intercept)*	− 0.595	0.751	0.627	0.428	0.55	[0.13, 2.40]	[0.13, 3.71]
MIRIC	1.292	0.482	7.170	0.007	3.64	[1.41, 9.37]	[1.32, 9.40]
Support for infant care							
Partner support only	− 1.306	0.785	2.764	0.096	0.27	[0.06, 1.26]	[0.06, 1.56]
Family support	− 1.677	0.789	4.514	0.034	0.19	[0.04, 0.88]	[0.04, 1.00]
Number of children							
≥ 3	1.019	0.446	5.219	0.022	2.77	[1.16, 6.65]	[1.15, 6.29]

MIRIC: Maternal Irritability in Response to Infant Crying. aOR: Adjusted Odds Ratio; CI: Confidence Interval. Internal validation was performed via non-parametric bootstrapping with 1000 resamples. Confidence intervals were derived using the percentile method (Bca).

## Discussion

The study showed that the prevalence of PPD was 23.5%, which is consistent with recent global reports for vulnerable populations. In this regard, Wang et al. (2021) documented a global prevalence of 17.22% (95% CI 16.00–18.51), being significantly higher in low- and middle-income countries^23^. In Latin America, research shows prevalences ranging from 13 to 21.7%, with Brazil being the country with the highest rates in the region^24^. In Colombia, the estimated national prevalence is 12.9% according to the 2010 National Demographic and Health Survey, with a higher frequency in urban areas (15.1%) compared to rural areas (6.8%)^25^. The findings of our study exceed these national figures, which may be explained by the specific characteristics of the population studied: mothers with preterm or low birth weight infants in kangaroo care programs, who face additional risk factors^26^.

The average age of the participants (27 years) and the higher proportion of young women with postpartum depression are consistent with recent evidence. A US study of more than 1.1 million mothers worldwide identified that mothers under the age of 25 are at the highest risk of postpartum depression^27^. In contrast to our findings, evidence from a large U.S. cohort indicates that older maternal age is associated with a higher risk of postpartum depression. Specifically, women aged 40–44 years showed a 3.72-fold increased risk (OR: 3.72; 95% CI 2.15–6.41) compared with women aged 30–35 years^28^. This discrepancy may reflect differences in population characteristics, reproductive trajectories, and contextual factors, underscoring the importance of considering setting-specific determinants when interpreting maternal age as a risk factor^29^.

Recent studies indicate that economic hardship is the strongest predictor of postpartum depression^30,31^. In our study, the predominant population was of middle and low socioeconomic income. In turn, Mexican research confirms that women in below-median income groups have the highest prevalence of postpartum depression (22%) compared to middle- and high-income groups^32^. Income emerges as the strongest predictor among all social status indices, surpassing even education and occupational prestige^33^.

The findings relating to a higher number of pregnancies, vaginal deliveries, and live births to PPD are differential findings concerning the literature and what is presented in high-income countries. A Japanese study of 1509 patients showed that primiparas have a higher risk of depressive symptoms (22.2%) compared to multiparas (11.6%) (OR: 1.65; 95% CI 1.31–2.06)^34^. On the other hand, Spanish research with 2990 postpartum women confirmed that primiparas are 1.6 times more likely (OR: 1.60; 95% CI 1.35–1.89) to have feelings of sadness and 1.65 times more likely to have depressive symptoms^34^. This difference in results can be explained by the specific characteristics of our population: mothers who participate in the kangaroo care program may experience greater anxiety when faced with neonatal complications due to their previous experience of childbirth. Furthermore, in Colombia, mothers give birth at a younger age than in the countries mentioned above, and families have a higher average number of children^35^.

Although our study found only marginal statistical differences according to mode of delivery, recent evidence suggests significant associations. A 2019 meta-analysis reported that cesarean delivery is associated with a higher risk of postpartum depression (OR: 2.0; 95% CI 1.35–2.96) compared to vaginal birth^36^. In turn, an Iranian study of 300 postpartum women documented that 13.3% of women who had cesarean sections developed depression, compared to 7.1% of those who had vaginal births. However, the heterogeneity between studies suggests that other moderate factors, such as the experience of kangaroo care, may influence this association^37^.

The most significant finding of our study was the association between maternal irritability in response to infant crying and postpartum depression (β = 1.14, *p* = 0.01). This relationship is well documented in recent literature. A longitudinal study of 587 mothers showed that maternal reports of inconsolable infant crying for more than 20 min per day were associated with an OR of 4.0 (95% CI 2.0–8.1) for postpartum depression^38^. Oberlander et al. (2017) reported that exposure to infant crying predicts dynamic fluctuations in maternal mental health, with effects accumulating over hours^39^. Specifically, when crying was above average for 8 h, mothers reported subsequent increases in depressive symptoms. Recent neurological research demonstrates that depressed mothers show reduced neural activation in regions related to emotional response and regulation when they hear their babies cry^39^.

The negative association between caregiving and postpartum depression (β = − 0.47, *p* = 0.04) suggests that caregiving support acts as a protective factor. A Korean study during the COVID-19 pandemic identified that paternal involvement in infant care significantly reduces the risk of postpartum depression^40^. Research on mothers with infants in NICU showed that early parental engagement and early interactions reduce the risk of late postpartum depression^41^. This is particularly relevant in the context of kangaroo care, where family support is critical to the success of the program.

### Limitations

This study has the following limitations. First, its cross-sectional design prevents conclusions from being drawn about causality or temporal relationships between the identified risk factors and postpartum depression. Second, the use of non-probability sampling may limit the generalization of the results. Third, postpartum depression was assessed solely using the Edinburgh Postnatal Depression Scale, which, although validated, is a self-report instrument and not a diagnostic psychiatric interview, which could introduce classification bias. The sample size may limit statistical power, especially in multivariate analysis. This study did not analyze variables such as poor sleep quality and low maternal self-efficacy, work experience, workplace support and woman-centred care, which we recommend be considered in future studies. Finally, given that all participants were mothers of premature or low birth weight babies, the results are not applicable to all postpartum women.

## Conclusion

The study revealed the prevalence of postpartum depression of 23.5%. Psychoemotional factors, specifically maternal irritability in response to infant crying, were associated with an increased risk of postpartum depression, while support in caring for the infant showed a protective effect. Similarly, multiparity, a higher number of vaginal births and a higher number of live births were associated with an increased risk. These results highlight the importance of considering context-specific factors and psycho-emotional dynamics in the identification and prevention of postpartum depression in kangaroo care settings.

### Recommendations

It is recommended that systematic screening protocols for postpartum depression be implemented in all kangaroo care programs, using the EPDS with culturally validated cut-off points. It is essential to develop specific interventions aimed at improving maternal skills for managing infant crying and strengthening family support networks. Finally, maternal mental health assessments need to be integrated into clinical practice guidelines for kangaroo care programs, with clear protocols for referral to specialized mental health services.

## Supplementary Information

Below is the link to the electronic supplementary material.

Supplementary Material 1

## Data Availability

The datasets generated and/or analyzed during the current study are not publicly available due to promises of participant anonymity and confidentiality but are available from the corresponding author upon reasonable request.

## Abbreviations

PPD: Postpartum depression
DSM-5: Mental disorder diagnose and statistical manual
EPDS: Edinburgh’s postpartum depression scale
