# Supplementary material for: Factors associated with postpartum depression in mothers of the kangaroo mother care program in Southern Colombia: a cross-sectional study
Source: Sci Rep. 2026 Jun 12;16:18262. doi: 10.1038/s41598-026-45420-5 (PMC13260941; doi:10.1038/s41598-026-45420-5)
Supplement: Supplementary file 1 — Supplementary Material 1 [file 41598_2026_45420_MOESM1_ESM.docx]

**Supplementary Tables**

The following tables present the analytical procedures that complement the information provided in the main text of the manuscript, Factors Associated with Postpartum Depression in Mothers of the Kangaroo Mother Care Program in Southern Colombia: A Cross-Sectional Study. The data supplied in Tables 1 and 2 correspond to the socio-demographic, gynaeco-obstetric, and psycho-emotional variables that did not reach statistical significance in the Pearson’s chi-squared (*χ²*) analysis

Supplementary Table S1. Socio-demographic factors of mothers attending the kangaroo program

| **Factors** | **Depression Indicators** | | | | ***χ²*** | | ***p-*value** | |  |
| --- | --- | --- | --- | --- | --- | --- | --- | --- | --- |
|  | **No** | **%** | **Si** | **%** | |  | |  | |
| Residence |  |  |  |  | |  | |  | |
| Urban | 74 | 77.9 | 21 | 22.1 | | 0.35 | | .553 | |
| Rural | 33 | 73.3 | 12 | 26,7 | |  |  |  |  |
| SES |  |  |  |  | |  | |  | |
| Low | 99 | 76.2 | 31 | 23.8 | | 0.08 | | .782 | |
| Medium | 8 | 80.0 | 2 | 20.0 | |  |  |  |  |
| Cohabiting |  |  |  |  | |  | |  | |
| No | 102 | 76.7 | 31 | 23.3 | | 0.10 | | .749 | |
| Yes | 5 | 71.4 | 2 | 28.6 | |  |  |  |  |
| Occupation |  |  |  |  | |  | |  | |
| No | 81 | 76.4 | 25 | 23.6 | | 0.00 | | .995 | |
| Yes | 26 | 76.5 | 8 | 23.5 | |  |  |  |  |
| Family support |  |  |  |  | |  | |  | |
| No | 68 | 80.0 | 17 | 20.0 | | 1.53 | | .216 | |
| Yes | 39 | 70.9 | 16 | 29.1 | |  |  |  |  |
| Schooling |  |  |  |  | |  | |  | |
| High school graduate | 69 | 71.9 | 27 | 28.1 | | 5.32 | | .070 | |
| Technical degree | 26 | 92.9 | 2 | 7.1 | |  |  |  |  |
| University degree | 12 | 75.0 | 4 | 25.0 | |  |  |  |  |

Note: SES: Socio-economic status. The statistic for comparing the factors was Pearson’s chi-squared (*χ²*) test

Supplementary Table S2. Gynaeco-obstetric and Psycho-emotional factors of mothers attending the kangaroo program

| **Factors** | **Depression Indicators** | | | | ***χ²*** | ***p*-value** |
| --- | --- | --- | --- | --- | --- | --- |
|  | **No** | **%** | **Si** | **%** |  |  |
| Abortions |  |  |  |  |  |  |
| No | 86 | 78.2 | 24 | 21.8 | 0.88 | .349 |
| Yes | 21 | 70.0 | 9 | 30.0 |  |  |
| Number controls prenatal |  |  |  |  |  |  |
| Less than six | 39 | 72.2 | 15 | 27.8 | 0.86 | .353 |
| Equal or more than six | 68 | 79.1 | 18 | 20.9 |  |  |
| Gestational age |  |  |  |  |  |  |
| <28 weeks | 2 | 100 | 0 | 0.0 | 0.81 | .848 |
| 28 to 32 weeks | 16 | 72.7 | 6 | 27.3 |  |  |
| >32 to < 37 weeks | 67 | 77.0 | 20 | 23.0 |  |  |
| Equal > 37 weeks | 22 | 75.9 | 7 | 24.1 |  |  |
| Type of delivery |  |  |  | . |  |  |
| Vaginal | 38 | 79.2 | 10 | 20.8 | 0.30 | .581 |
| Caesarean | 69 | 75.0 | 23 | 25.0 |  |  |
| History of illness |  |  |  |  |  |  |
| No | 91 | 76.5 | 28 | 23.5 | 0.00 | .978 |
| Yes | 16 | 76.2 | 5 | 23.8 |  |  |
| Pregnancy-associated illness |  |  |  |  |  |  |
| No | 34 | 87.2 | 5 | 12.8 | 3.47 | 0.063 |
| Yes | 73 | 72.3 | 28 | 27.7 |  |  |
| Breastfeeding difficulty |  |  |  |  |  |  |
| No | 87 | 79.1 | 23 | 20.9 | 2.02 | .155 |
| Yes | 20 | 66.7 | 10 | 33.3 |  |  |
| Perinatal complication |  |  |  |  |  |  |
| No | 12 | 92.3 | 1 | 7.7 | 2.01 | .157 |
| Yes | 95 | 74.8 | 32 | 25.2 |  |  |
| Desired pregnancy |  |  |  |  |  |  |
| No | 87 | 79.8 | 22 | 20.2 | 3.14 | .077 |
| Yes | 20 | 64.5 | 11 | 35.5 |  |  |
| Fulfils maternal role |  |  |  |  |  |  |
| No | 105 | 77.2 | 31 | 22.8 | 1.60 | .206 |
| Yes | 2 | 50.0 | 2 | 50.0 |  |  |

Note: The statistic for comparing the factors was Pearson’s chi-squared (*χ²*) test

**Model Selection Procedure**

The statistical process for determining the final multivariable logistic regression model is detailed in Table S3, illustrating a stepwise backward elimination approach. The procedure commenced with a full model (*M_0_*) incorporating all theoretically relevant and bivariately significant predictors, including socio-demographic, gynaeco-obstetric, and psycho-emotional factors. Variables that failed to contribute significantly to the model's explanatory power were removed sequentially based on the highest *p*-values from the Wald test, as their exclusion did not significantly alter the estimates of the remaining parameters.

The final parsimonious model (M_7_) was achieved by retaining variables with clinical relevance or statistical significance. In this terminal stage, maternal irritability in response to infant crying (MIRIC) emerged as the most robust predictor, with a significant adjusted Odds Ratio of 3.639 (*p* = .007; 95% CI [1.414, 9.369]). Regarding model fit and stability, it was observed that standard errors remained stable throughout the iterations, and the final selection was cross-validated against the Akaike Information Criterion (AIC) to ensure an optimal balance between model complexity and goodness-of-fit

Supplementary Table S3. Comparison of candidate models (*M_0_ –M_7_*) for factors associated with postpartum depression.

| **Model** | **Parameter** | **Estimate** | **Standard Error** | **Odds Ratio** | **Wald Test** | | **95% CI** | |
| --- | --- | --- | --- | --- | --- | --- | --- | --- |
|  |  |  |  |  | **Wald Statistic** | ***p*** | **Lower bound** | **Upper bound** |
| M₀ | (Intercept) | -0.057 | 1.546 | 0.945 | 0.001 | .971 | 0.046 | 19.560 |
|  | Age | 0.060 | 0.044 | 1.062 | 1.907 | .167 | 0.975 | 1.157 |
|  | SES | 0.357 | 0.948 | 1.429 | 0.142 | .707 | 0.223 | 9.156 |
|  | Schooling | -0.127 | 0.184 | 0.881 | 0.474 | .491 | 0.614 | 1.264 |
|  | Deliveries | 0.393 | 0.257 | 1.481 | 2.329 | .127 | 0.894 | 2.454 |
|  | Live births | -1.037 | 0.472 | 0.355 | 4.829 | .028 | 0.141 | 0.894 |
|  | Number of children (2) | 2.172 | 0.870 | 8.775 | 6.229 | .013 | 1.594 | 48.298 |
|  | Controls prenatal (2) | -0.368 | 0.474 | 0.692 | 0.602 | .438 | 0.273 | 1.754 |
|  | Abuse in pregnancy (1) | 1.512 | 1.386 | 4.537 | 1.191 | .275 | 0.300 | 68.587 |
|  | Partner support | -1.311 | 0.826 | 0.269 | 2.520 | .112 | 0.053 | 1.360 |
|  | Family support | -1.782 | 0.841 | 0.168 | 4.489 | .034 | 0.032 | 0.875 |
|  | MIRIC | 1.245 | 0.540 | 3.475 | 5.313 | .021 | 1.205 | 10.019 |
| M₁ | (Intercept) | -0.207 | 1.492 | 0.813 | 0.019 | .890 | 0.044 | 15.139 |
|  | Age | 0.062 | 0.043 | 1.064 | 2.023 | .155 | 0.977 | 1.158 |
|  | Schooling | -0.106 | 0.176 | 0.900 | 0.362 | .547 | 0.637 | 1.270 |
|  | Deliveries | 0.404 | 0.257 | 1.498 | 2.474 | .116 | 0.905 | 2.478 |
|  | Live births | -1.036 | 0.472 | 0.355 | 4.811 | .028 | 0.141 | 0.896 |
|  | Number of children (2) | 2.170 | 0.871 | 8.761 | 6.207 | .013 | 1.589 | 48.314 |
|  | Controls prenatal (2) | -0.369 | 0.474 | 0.691 | 0.607 | .436 | 0.273 | 1.749 |
|  | Abuse in pregnancy (1) | 1.506 | 1.384 | 4.507 | 1.183 | .277 | 0.299 | 67.925 |
|  | Partner support | -1.289 | 0.824 | 0.276 | 2.449 | .118 | 0.055 | 1.384 |
|  | Family support | -1.756 | 0.837 | 0.173 | 4.398 | .036 | 0.033 | 0.891 |
|  | MIRIC | 1.229 | 0.540 | 3.417 | 5.187 | .023 | 1.187 | 9.838 |
| M₂ | (Intercept) | -0.651 | 1.295 | 0.522 | 0.252 | .615 | 0.041 | 6.608 |
|  | Age | 0.060 | 0.043 | 1.062 | 1.915 | .166 | 0.975 | 1.156 |
|  | Deliveries | 0.410 | 0.256 | 1.508 | 2.576 | .109 | 0.913 | 2.489 |
|  | Live births | -1.017 | 0.471 | 0.362 | 4.672 | .031 | 0.144 | 0.910 |
|  | Number of children (2) | 2.181 | 0.870 | 8.853 | 6.287 | .012 | 1.610 | 48.689 |
|  | Controls prenatal (2) | -0.425 | 0.464 | 0.654 | 0.838 | .360 | 0.263 | 1.624 |
|  | MIRIC | 1.293 | 0.529 | 3.643 | 5.969 | .015 | 1.291 | 10.279 |
| M₃ | (Intercept) | -0.926 | 1.263 | 0.396 | 0.538 | .463 | 0.033 | 4.706 |
|  | Age | 0.058 | 0.043 | 1.059 | 1.805 | .179 | 0.974 | 1.153 |
|  | Deliveries | 0.376 | 0.252 | 1.457 | 2.219 | .136 | 0.888 | 2.389 |
|  | Live births | -0.949 | 0.459 | 0.387 | 4.271 | .039 | 0.157 | 0.952 |
|  | Number of children (2) | 2.093 | 0.856 | 8.106 | 5.972 | .015 | 1.513 | 43.414 |
|  | Abuse in pregnancy (1) | 1.567 | 1.335 | 4.790 | 1.377 | .241 | 0.350 | 65.577 |
|  | Partner support | -1.323 | 0.825 | 0.266 | 2.571 | .109 | 0.053 | 1.342 |
|  | Family support | -1.827 | 0.830 | 0.161 | 4.846 | .028 | 0.032 | 0.818 |
|  | MIRIC | 1.297 | 0.527 | 3.659 | 6.066 | .014 | 1.303 | 10.274 |
| M₄ | (Intercept) | -0.952 | 1.252 | 0.386 | 0.579 | .447 | 0.033 | 4.487 |
|  | Age | 0.057 | 0.042 | 1.058 | 1.829 | .176 | 0.975 | 1.149 |
|  | Deliveries | 0.398 | 0.249 | 1.489 | 2.554 | .110 | 0.914 | 2.425 |
|  | Live births | -0.961 | 0.452 | 0.382 | 4.517 | .034 | 0.158 | 0.928 |
|  | Number of children (2) | 2.163 | 0.847 | 8.695 | 6.517 | .011 | 1.652 | 45.755 |
|  | Partner support | -1.264 | 0.828 | 0.283 | 2.332 | .127 | 0.056 | 1.431 |
|  | Family support | -1.783 | 0.832 | 0.168 | 4.592 | .032 | 0.033 | 0.859 |
|  | MIRIC | 1.391 | 0.518 | 4.017 | 7.217 | .007 | 1.457 | 11.081 |
| M₅ | (Intercept) | 0.200 | 0.908 | 1.221 | 0.049 | .826 | 0.206 | 7.237 |
|  | Deliveries | 0.331 | 0.242 | 1.393 | 1.881 | .170 | 0.867 | 2.237 |
|  | Live births | -0.753 | 0.417 | 0.471 | 3.264 | .071 | 0.208 | 1.066 |
|  | Number of children (2) | 2.159 | 0.842 | 8.665 | 6.575 | .010 | 1.663 | 45.143 |
|  | Partner support | -1.262 | 0.806 | 0.283 | 2.451 | .117 | 0.058 | 1.374 |
|  | Family support | -1.738 | 0.815 | 0.176 | 4.552 | .033 | 0.036 | 0.868 |
|  | MIRIC | 1.398 | 0.508 | 4.045 | 7.558 | .006 | 1.494 | 10.957 |
| M₆ | (Intercept) | -0.013 | 0.892 | 0.987 | 2.118×10^-4^ | .988 | 0.172 | 5.667 |
|  | Live births | -0.415 | 0.331 | 0.660 | 1.579 | .209 | 0.345 | 1.262 |
|  | Number of children (2) | 1.819 | 0.779 | 6.165 | 5.448 | .020 | 1.339 | 28.398 |
|  | Partner support | -1.301 | 0.795 | 0.272 | 2.676 | .102 | 0.057 | 1.294 |
|  | Family support | -1.744 | 0.803 | 0.175 | 4.711 | .030 | 0.036 | 0.844 |
|  | MIRIC | 1.427 | 0.501 | 4.165 | 8.115 | .004 | 1.561 | 11.114 |
| M₇ | (Intercept) | -0.595 | 0.751 | 0.552 | 0.627 | .428 | 0.127 | 2.404 |
|  | Number of children (2) | 1.019 | 0.446 | 2.772 | 5.219 | .022 | 1.156 | 6.646 |
|  | Partner support | -1.306 | 0.785 | 0.271 | 2.764 | .096 | 0.058 | 1.263 |
|  | Family support | -1.677 | 0.789 | 0.187 | 4.514 | .034 | 0.040 | 0.878 |
|  | MIRIC | 1.292 | 0.482 | 3.639 | 7.170 | .007 | 1.414 | 9.369 |

Note: MIRIC: Maternal Irritability in Response to Infant Crying. aOR: Adjusted Odds Ratio; CI: Confidence Interval

**Collinearity Diagnostics for the Multivariable Model**

To ensure the stability and reliability of the final regression coefficients, multicollinearity diagnostics were performed for all predictors included in the model. All variables exhibited Variance Inflation Factor (VIF) values near 1 (ranging from 1.052 to 1.130), which is well below the conservative threshold of 5 (or even the more liberal threshold of 10). Regarding tolerance levels, values ranged from 0.885 to 0.951, significantly exceeding the common cut-off of 0.10. In conclusion, these results indicate a lack of multicollinearity among the predictors. Specifically MIRIC, number of children, and support for infant care, confirming that each variable provides unique and stable information to the multivariable model

Supplementary Table S4. Collinearity diagnostics for the final multivariable regression model.

| **Predictor** | **Tolerance** | **VIF** |
| --- | --- | --- |
| MIRIC | 0.923 | 1.083 |
| Number of children | 0.951 | 1.052 |
| Support for infant care | 0.885 | 1.130 |

Note: MIRIC: Maternal Irritability in Response to Infant Crying. VIF: Variance Inflation Factor
